# No Matter When or Where: Addressing the Need for Continuous Family Planning Services During Shocks and Stressors

**DOI:** 10.9745/GHSP-D-24-00124

**Published:** 2024-12-20

**Authors:** Sarah Rich, Lily Jacobi, Nesrine Talbi, Ashley Wolfington, Kelly McDonald

**Affiliations:** a Promoting Results and Outcomes through Policy and Economic Levers Adapt, New York, NY, USA.; b Women’s Refugee Commission, New York, NY, USA.; c FP2030, Bordeaux, France.; d Promoting Results and Outcomes through Policy and Economic Levers Adapt, Ocean Grove, NJ, USA.; e Promoting Results and Outcomes through Policy and Economic Levers Adapt, Washington, DC, USA.

## Abstract

Global progress on meeting family planning needs is threatened by worsening, intersecting crises. We call on global, national, and local partners to strengthen emergency preparedness to facilitate continuous family planning services, no matter when or where they are needed, to support sexual and reproductive health.

## Key Messages

- Global progress on meeting family planning (FP) needs is threatened by worsening, intersecting crises that disrupt health systems and disproportionately affect women, girls, and historically marginalized people. No country is immune to climate change risks or infectious disease outbreaks, and the number, intensity, and impacts of conflicts are growing.
- People need continuous access to essential health services, including FP services. Evidence demonstrates robust demand for FP services during and after diverse crises. Yet FP availability, access, and quality remain limited and uneven during crises. Disruptions in FP services undercut the efficacy of health systems and policies and derail advancements made during stable times.
- Emergency preparedness facilitates quicker access to services when crises occur and saves money. However, preparing for continuous essential health services, including FP, is overlooked and deprioritized in existing funding streams and implementation mechanisms. We provide recommendations for global, national, and local partners–including governments, donors, and development and humanitarian agencies–to strengthen emergency preparedness for FP. Ensuring ongoing FP services during shocks and stressors saves lives, fulfills human rights, and advances health care for all.

## INTRODUCTION

Global progress on meeting voluntary family planning (FP) needs has been advancing. In 2022, 371 million women worldwide were using a modern contraceptive method, marking an increase of 87 million over the past decade, with expanded use observed in all regions.^1^ However, advances have been uneven across and within countries and demographic groups. Moreover, this hard-fought progress is threatened everywhere by worsening, intersecting crises that disrupt health systems and disproportionately affect women, girls, and historically marginalized people. No region or setting is immune to the risks posed by climate change and infectious disease outbreaks, and the growing number and severity of conflicts have drained resources for health in many countries.^2^^,^^3^

The impacts of crises today are wide-reaching. In 2024, an estimated 300 million individuals will need humanitarian assistance,^2^ including 75 million women and girls of reproductive age. If this entire population were consolidated into a single country, it would rank as the fourth largest globally.^4^ Additionally, the number of forcibly displaced individuals each year has been on the rise for over 2 decades.^2^ The challenges extend beyond large-scale emergencies that require a global humanitarian response. More than half the world’s countries are at medium, high, or very high risk of crisis,^5^ and all countries will inevitably face shocks and stressors.

When crises occur, people need continued access to essential health care, including sexual and reproductive health (SRH) services. Yet during crises—from conflicts to natural disasters to infectious disease outbreaks—availability of and access to quality FP services remains limited and uneven.^6^ In this article, we focus on 1 service, FP, which is often disrupted in times of crises, undercutting the efficacy of health systems and policies and derailing advancements made during stable times.

Strengthening emergency preparedness can mitigate the impacts of crises. While calls for better preparedness are not new, rising global instability and crises make it increasingly urgent. Momentum to strengthen preparedness is starting to build. Governments worldwide are making commitments to emergency preparedness for FP. Climate change activism now recognizes that weather emergencies have an outsized impact on women and girls and increasingly includes efforts to ensure access to SRH services.

However, preparing for continuous FP services in the face of shocks and stressors remains overlooked and deprioritized in existing funding streams and implementation mechanisms. Preparedness is often seen as someone else’s job. In truth, everyone has a role to play in better equipping communities and health systems to withstand the inevitable shocks and continue to deliver essential FP services during crises.

Everyone has a role to play in better equipping communities and health systems to withstand the inevitable shocks and continue to deliver essential FP services during crises.

We call on global, national, and local partners to strategically invest in emergency preparedness to ensure continuous FP services and foster new, multisectoral partnerships to strengthen preparedness at all levels and across all settings. Ensuring ongoing FP services during shocks and stressors is critical to save lives, fulfill the human rights originally enshrined at the International Conference on Population and Development in 1994,^7^ and continue to make progress toward health care for all.

In this commentary, we review evidence on the need for ongoing FP services during shocks and stressors, outline the benefits of and challenges to strengthening preparedness, discuss progress made to date, and offer recommendations for action to catalyze transformative changes in emergency preparedness for FP. The recommendations are aimed at stakeholders working across humanitarian, development, and fragile settings: governments, including health and disaster risk management stakeholders; donors; multilateral institutions, such as the United Nations Population Fund (UNFPA) and the World Health Organization; local, national, and international implementing agencies; researchers and academic institutions; and civil society organizations and advocates.

## THE NEED FOR CONTINUOUS FAMILY PLANNING SERVICES DURING CRISES

During crises, health risks, including maternal morbidity and mortality and gender-based violence (GBV), increase. Over 60% of preventable maternal deaths occur in countries that host fragile, conflict-affected, and vulnerable settings.^8^ At the same time, FP services are often disrupted during shocks and stressors, leaving people affected by crises with gaps in access to FP services.

FP is an essential, lifesaving component of primary health care and part of the minimum standards of care in crisis-affected settings. The Minimum Initial Service Package (MISP) for SRH, recognized as the global standard in acute emergency response, includes the prevention of unintended pregnancies as 1 of 6 priority objectives to prevent morbidity and mortality; the other health areas included in the MISP are maternal and newborn health care, GBV prevention and response, and prevention of and treatment of HIV and sexually transmitted infections.^9^ The objectives and activities in the MISP are integrated into the Sphere Standards, which delineate humanitarian principles and minimum standards in 4 lifesaving areas, including health.^10^ The MISP objectives address the main causes of excess maternal and newborn mortality and align with most health systems’ approaches to primary health care and reproductive, maternal, newborn, child, and adolescent health services. The MISP is intended to be implemented by a consortium of government, development, and humanitarian actors to cover the lifesaving needs of the affected population. As the Interagency Working Group on Reproductive Health in Crises noted^11^:

*The MISP acknowledges that humanitarian actors will need to navigate legal barriers to abortion in some contexts. The MISP is meant to be implemented in a consortium of many actors in crises. Not all actors need to provide safe abortion care. This is true of all MISP interventions.*

The MISP calls for ensuring availability of a range of long-acting and short-acting FP methods, including emergency contraception, to meet demand; providing information and contraceptive counseling that emphasizes informed choice and consent, effectiveness, privacy and confidentiality, equity, and nondiscrimination; and ensuring the community is aware of the availability of contraceptives for women, adolescents, and men. These services, along with services for maternal and newborn health, GBV, and HIV and sexually transmitted infections, must be made available from the onset of every crisis. Service delivery should be expanded to include comprehensive FP services as soon as possible as an acute crisis stabilizes, in alignment with the *Inter-Agency Field Manual (IAFM) on Reproductive Health in Humanitarian Settings*.^12^

Demand for FP services has consistently been documented during crises. Across diverse contexts, 30%–40% of women experiencing displacement did not want to become pregnant in the next 2 years, and 12%–35% wanted to limit the number of pregnancies.^13^ In some cases, the proportion of women who want to prevent pregnancy can be even higher. For example, nearly three-quarters of pregnant Syrian refugee women surveyed in Lebanon wished to prevent future pregnancy, and more than one-half did not desire their current pregnancy.^14^ Evidence from the COVID-19 pandemic has reinforced that women and adolescents want and need FP during and in the aftermath of infectious disease outbreaks.^15^ Similar data documented rebounds in demand for FP following service disruptions during the Ebola outbreak of 2014.^16^

Evidence shows that providing quality FP services during crises is feasible and demonstrates that people affected by crises use FP services when they are available and of adequate quality.^17^ Strong uptake of FP has been observed in diverse humanitarian settings worldwide, including in natural disasters like the aftermath of the earthquake in Nepal in 2015,^18^ acute conflicts such as the war in Yemen,^19^ protracted conflict settings like the eastern region of the Democratic Republic of the Congo^20^^,^^21^ and northern Uganda,^22^ and after infectious disease outbreaks such as Ebola in West Africa.^16^

However, FP services are not consistently available during crises. Even when available, specific gaps often persist. These include the availability of a range of contraceptive methods (particularly long-acting reversible contraception and emergency contraception), barriers to access for adolescents and members of other historically marginalized groups, gaps in availability of FP commodities, and poor data collection and use.^5^

## THE VALUE-ADD OF INVESTING IN PREPAREDNESS

With rising crises worldwide and growing numbers of people who are affected, all countries face risks and need to mitigate and respond to their impacts. Emerging evidence shows that investing in preparedness not only enables a faster response when emergencies occur but also saves money. One study of emergency preparedness investments in Chad, Madagascar, and Pakistan found that all of the investments saved money and/or time.^23^ A follow-up study, which calculated longer-term savings and examined additional preparedness investments, found that for every US$1 invested in emergency preparedness, US$1.5 was saved in response and that preparedness sped up response time by 2 weeks on average.^24^ A study examining the impact of investments in capacity development as part of preparedness in Ethiopia and the Philippines found an average financial return on investment of £2.84 per £1 invested.^25^ Another study examining preparedness investments in Indonesia, Madagascar, and Nepal similarly found that all of the investments saved time, money, or both.^26^ While these studies were not focused specifically on FP, they point to the broad potential and cost-effectiveness of preparedness efforts to mitigate service disruptions during crises.

Emerging evidence shows that investing in preparedness not only enables a faster response when emergencies occur but also saves money.

Ensuring continuous access to FP services is also critical to achieving the national and international commitments that countries and other stakeholders have made. Given increases in the number of people affected and potential for backslide when crises occur, the individuals who are affected by shocks and stressors cannot be ignored on the journey to achieving the Sustainable Development Goals, universal health coverage, and FP2030 commitments.

Moreover, strengthening preparedness benefits health systems during stable times and crises alike. A literature review of the evidence base for factors that make a health system resilient identified themes related to strong governance and leadership, integrated approaches to health service delivery, a well-trained health workforce including community health workers, adequate health system financing, community engagement, and equity in reaching all segments of the population. These factors not only facilitate continuous access to health services during crises but also strengthen health systems during stable times.^27^

## CHALLENGES TO STRENGTHENING PREPAREDNESS

When decision-makers do not plan for and take action to ensure continuity of FP services during shocks and stressors, women and girls often lose access to these essential health services.^28^^,^^29^ Yet persistent silos in humanitarian and development funding, policymaking, coordination mechanisms, and programming lead stakeholders to overlook and deprioritize preparedness. Development actors (local and international alike) often do not incorporate emergency preparedness within their remit, while humanitarian actors are not always present before a crisis occurs. Even governments and organizations with dual humanitarian and development mandates often have isolated programming across these spheres.^30^ Factors like short-term funding cycles for humanitarian response, along with long-standing patterns of working, further limit opportunities to collaborate.^31^ These dynamics ultimately impede continuity of access to FP services as crises occur, subside, and recur.

These silos do not reflect the dynamic reality that many countries face, yet stakeholders across the spectrum find challenges in identifying earmarked funding and opportunities to support preparedness. Donors report that within the existing financing structures, none seem to encompass FP preparedness (unpublished data). Enduring splits across development, humanitarian, and climate-related funding streams and implementation mechanisms continue to relegate preparedness and other work in the humanitarian-development-peace nexus to the sidelines.

Readiness to provide FP services during shocks and stressors calls for strategic investments, including coordinated and complementary actions. Coordination requires not only commitment but also time and funding, especially to bridge the diverse priorities, systems, and even language across the humanitarian and development sectors. Actors working across these spheres often struggle to effectively harness their strengths to achieve shared goals, including preparedness. Forging these networks at the start of an acute emergency is too late—the work of building a movement for FP resilience occurs during the preparedness phase.

## THE MOMENTUM TO STRENGTHEN PREPAREDNESS IS GROWING

The 2016 World Humanitarian Summit identified bridging the divide between the humanitarian and development sectors as a top priority. The New Way of Working provides a framework to leverage the comparative advantages of humanitarian and development actors toward commonly agreed collective outcomes over multiyear timeframes.^32^ Since then, the international aid community has recognized the importance of integrating peacebuilding actors, forming the “triple nexus,” or the humanitarian-development-peace nexus. The nexus recognizes that countries do not make linear transitions across stability, development, fragility, and crises but can experience degrees of each and fluctuate back and forth.

Momentum toward strengthening preparedness for FP specifically has also grown over the past several years. For example, the Family Planning High Impact Practices published “Family Planning in Humanitarian Settings: A Strategic Planning Guide,” highlighting to a broad audience the importance of providing FP services to people affected by crises and providing guidance on critical actions during preparedness, response, and recovery.

Momentum toward strengthening preparedness for FP specifically has also grown over the past several years.

Investments from major donors, including some influential bilateral funders, also reflect a growing commitment to strengthening preparedness. The Australian government has long invested in emergency preparedness and response initiatives for SRH in the Asia-Pacific region, including the SPRINT Initiative, implemented by the International Planned Parenthood Federation, and the Regional Prepositioning Initiative, managed by UNFPA. The U.S. Agency for International Development has 2 current investments, the Momentum Integrated Health Resilience and Promoting Results and Outcomes through Policy and Economic Levers Adapt projects, that dedicate development funds toward strengthening resilience for FP, including preparedness. For example, the MOMENTUM Integrated Health Resilience project developed an FP Resilience Checklist to assess the extent to which FP efforts integrate interventions to strengthen resilience of individuals, couples, communities, and facilities. The Promoting Results and Outcomes through Policy and Economic Levers Adapt project developed a policy resilience scorecard to assess whether existing policy frameworks promote resilience for FP/reproductive health. These and other investments reflect growing donor commitment to preparedness for FP.

Moreover, FP2030, the flagship global partnership to advance access to FP globally, has devoted increasing resources to strengthening preparedness and response, including hiring dedicated staff. FP2030 launched its updated Emergency Preparedness and Response strategy in 2022 and is establishing a center of excellence to foster cross-country and cross-regional learnings; provide technical assistance; create an online resource center; and advance the national, regional, and global preparedness and response agenda. FP2030 has also supported governments to include preparedness and response in their national commitments. As of July 2024, 76% of FP2030 commitment-making countries (28 of 37 countries) had integrated emergency preparedness and response in their commitments. This is a marked increase compared to 2017, when only 1 country made a specific commitment to preparedness and response.

Additionally, between 2021 and 2024, UNFPA supported countries across multiple regions, including East and Southern Africa, West and Central Africa, Arab States, and Eastern Europe and Central Asia, to conduct MISP Readiness Assessments. These multistakeholder processes assess SRH preparedness and bring together stakeholders across different sectors, including development, humanitarian, protection, health, and disaster management, to develop action plans in each country to address gaps. The investment in MISP Readiness Assessments signals a commitment to preparedness from UNFPA and has helped to operationalize working in the humanitarian-development-peace nexus across numerous countries.

Global health stakeholders are increasingly vocal about the importance of preparedness. Atul Gawande, Assistant Administrator for Global Health at the U.S. Agency for International Development, wrote in *The New York Times* that during his tenure, the continuous stream of overlapping emergencies, from COVID-19 to the war in Ukraine, has diverted attention from longer-term global health goals. He noted that “longer-range investment in local preparedness for such events…could reduce the threat these crises pose and even reduce dependence on foreign aid to weather them.”^33^ Gawande’s article is an example of a high-level stakeholder—outside of the humanitarian sector—making the case that preparedness is critical to achieving global health and development goals.

## EVIDENCE TO DATE ON WHAT WORKS TO STRENGTHEN FAMILY PLANNING PREPAREDNESS

Many of the recent investments in preparedness are too new to provide rigorous evidence on what works to strengthen preparedness for continuous FP services during crises. However, learning has been documented from the longer-standing investments in the Asia-Pacific region, particularly on supply chain preparedness. For example, the Regional Prepositioning Initiative supported supply chain preparedness to enable faster availability of supplies—including FP commodities—during crises in the region, reporting both time and cost savings across 69 emergencies in 15 countries.^34^ In addition to prepositioning supplies, other supply chain preparedness activities have also improved availability of supplies during crises, including developing long-term agreements with suppliers, improving supply chain data visibility, and creating feedback loops to strengthen accountability for the quality of supplies and performance of suppliers.^35^ Preparedness can also support more inclusive SRH responses. For example, under the SPRINT Initiative, the Family Planning Association of Sri Lanka added representatives from organizations representing people with disabilities and people with diverse sexual orientation, gender identity, gender expression, and sex characteristics to the project steering committee, leading to developing information, education, and communications materials for the MISP, including FP, in sign language and braille and distributing dignity kits, which support hygiene with items like soap and menstrual health products, to transgender individuals, including during 2018 floods.^35^ A range of other preparedness strategies to strengthen policies, coordination, training, and other areas have been undertaken and documented to date, such as those included in *Ready to Save Lives: A Preparedness Toolkit for Sexual and Reproductive Health Care in Emergencies*, pointing to the feasibility of and potential for impact of implementing diverse preparedness actions.

Additionally, learning from effective measures to mitigate FP disruptions in emergency response, particularly during COVID-19, can inform the development of future preparedness efforts. Service delivery adaptations implemented during COVID-19, like telehealth and leveraging digital solutions; task-shifting/sharing and community-based provision of services; self-care; and coordinated supply chain actions, such as redistributing supplies between districts and facilities according to demand and availability, helped mitigate service disruptions.^28^^,^^36^^,^^37^ One study examining the resilience of FP services across 70 countries during the COVID-19 pandemic found that a robust enabling environment for FP was strongly associated with FP resilience during the pandemic.^38^ Another article similarly points to the importance of strong policy and governance, including ensuring that SRH coordination mechanisms (under which FP services are coordinated) are activated; that national and subnational policies explicitly state that SRH services are essential during crises; and that advocacy and accountability mechanisms elevate evidence of the impacts of failing to prioritize SRH services.^39^ This article focused on SRH broadly with a particular emphasis on FP. Learning from Sierra Leone also found that strong government leadership, leveraging lessons learned from the Ebola epidemic, supported ongoing service delivery.^40^ Overall, learning from the COVID-19 pandemic underlines the importance of strong policies, governance, leadership, and adaptive service delivery and supply chain mechanisms in mitigating service disruptions during crises; these lessons should be applied to ongoing preparedness efforts.

Learning from effective measures to mitigate FP disruptions in emergency response can inform the development of future preparedness efforts.

## RECOMMENDATIONS TO ENSURE CONTINUOUS FAMILY PLANNING SERVICES THROUGH SHOCKS AND STRESSORS

The global, national, and local humanitarian and development communities must continue to build on the momentum to better prepare for uninterrupted FP services at all levels and across all settings. To strengthen preparedness to provide ongoing FP services through shocks and stressors, stakeholders must systematically integrate crisis preparedness and risk management into FP and other SRH policies, financing, coordination mechanisms, and programs while also ensuring that FP preparedness and response are integrated into ongoing disaster risk management and climate resilience policies, financing, coordination mechanisms, and programs.

Specific recommended actions include the following.

- **Demonstrate high-level political commitment** by integrating emergency preparedness and response into FP commitments and including voluntary, rights-based FP in disaster risk management and climate resilience commitments.
- **Integrate preparedness for FP into national and subnational SRH, FP, disaster risk management, and emergency preparedness and response policies** while developing preparedness strategies that outline objectives, plans, targets, and indicators for FP service delivery when shocks and stressors occur.
- **Incorporate FP preparedness into global and national normative guidance** to establish it as a standard practice.
- **Allocate dedicated, multiyear funding for FP preparedness** via government, donor, and other domestic and global financing mechanisms to develop and implement preparedness strategies, such as the ones listed here.
- **Strengthen coordination** by integrating emergency preparedness topics and experts into standing national committees and working groups, such as national FP or SRH technical working groups and supply chain or commodity security working groups, and working in partnership with health clusters and SRH subclusters (which coordinate FP implementation during emergencies) when activated.
- **Create feedback mechanisms to ensure that learning from emergency response informs FP preparedness**. For example, data on the extent to which quality FP services were available and accessible in a given response should feed into and strengthen future preparedness efforts.
- **Strengthen workforce readiness** to provide FP services during crises by integrating (as legally appropriate) the MISP for SRH, which includes FP as a pillar, into national training curriculums for health providers and developing surge rosters.
- **Adopt and scale evidence-based service delivery modalities,** like the High Impact Practices for FP, that facilitate access to FP services in both stable times and crises alike, such as community health workers, postpartum and postabortion FP services, and mobile outreach.^41^
- **Strengthen supply chain preparedness** by developing supply chain contingency plans, prepositioning or stockpiling supplies where frequent crises occur, improving supply chain data visibility, and training supply chain managers on emergency supply chain management, including for FP commodities.
- **Hold governments and other duty-bearers accountable** to existing policies, strategies, and commitments by supporting civil society organizations to publicize relevant commitments and call out gaps in achieving them.
- **Conduct rigorous monitoring and evaluation** of preparedness efforts to continue building the evidence base on what works to enable continuous essential health services, including FP, during crises.
- **Include young people, people with disabilities, people with diverse sexual orientations, gender identities, gender expressions, and sex characteristics, and other historically marginalized groups** in the development and implementation of the above activities to ensure their priorities are reflected and their needs are met.

Governments, donors, humanitarian and development agencies, civil society organizations, and communities have a role to play in ensuring continuity of essential services, including FP, from stable times through shocks and stressors.^42^ As stewards of their national health programs, governments must lead the way on advancing policies, programs, and financing that prepare for shocks and stressors to enable continuous access to essential health services, including FP. Local and international development actors, including civil society organizations, that have long-standing presence and relationships at the country level are well-positioned to partner with governments to strengthen pre-crisis emergency preparedness. Governments and development actors can partner with humanitarian agencies to leverage their respective expertise in emergency preparedness and response. As local communities are the first responders when crises occur, and local organizations are particularly well placed to adapt and respond quickly when shocks occur, they must be centered and engaged in preparedness efforts.

As noted previously, the FP HIPs Family Planning in Humanitarian Settings: A Strategic Planning Guide identifies a range of actions from preparedness to response and recovery that improve continuous access to FP. Additionally, several broader SRH-related tools exist to guide this work (elements of some of these tools related to provision of safe abortion care may not be feasible for specific actors or in specific legal contexts). These tools include: *Ready to Save Lives: A Preparedness Toolkit for Sexual and Reproductive Health Care in Emergencies,* which brings together existing learning and guidance for SRH preparedness; MISP Readiness Assessments that have been completed in many countries to identify context-specific activities that should be implemented; and the *Facilitator’s Kit: Community Preparedness for Sexual and Reproductive Health and Gender*, which offers tools for government agencies at the subnational level to support local stakeholders to strengthen community capacity to prepare for crises.

Investing in emergency preparedness preserves progress made during stable times and facilitates continuous access to sustainable FP services for people who are affected by shocks and stressors. Partners working across all settings—whether stable, fragile, or crisis-affected—must take action to better prepare health systems to deliver services, including FP, to all—no matter who they are, where they live, or when they need them.

## Notes

**Peer Reviewed**

**First Published Online:** December 5, 2024.
